# Temporal contrast effects in human speech perception are immune to selective attention

**DOI:** 10.1038/s41598-020-62613-8

**Published:** 2020-03-27

**Authors:** Hans Rutger Bosker, Matthias J. Sjerps, Eva Reinisch

**Affiliations:** 1 Max Planck Institute for Psycholinguistics, PO Box 310, 6500 AH Nijmegen, The Netherlands; 2 Donders Institute for Brain, Cognition and Behaviour, Radboud University, Nijmegen, the Netherlands; 3 Institute of Phonetics and Speech Processing, Ludwig Maximilian University Munich, Munich, Germany; 4 Acoustics Research Institute, Austrian Academy of Sciences, Vienna, Austria

**Keywords:** Attention, Human behaviour

## Abstract

Two fundamental properties of perception are selective attention and perceptual contrast, but how these two processes interact remains unknown. Does an attended stimulus history exert a larger contrastive influence on the perception of a following target than unattended stimuli? Dutch listeners categorized target sounds with a reduced prefix “ge-” marking tense (e.g., ambiguous between *gegaan-gaan* “gone-go”). In ‘single talker’ Experiments 1–2, participants perceived the reduced syllable (reporting *gegaan*) when the target was heard after a fast sentence, but not after a slow sentence (reporting *gaan*). In ‘selective attention’ Experiments 3–5, participants listened to two simultaneous sentences from two different talkers, followed by the same target sounds, with instructions to attend only one of the two talkers. Critically, the speech rates of attended *and* unattended talkers were found to equally influence target perception – even when participants could watch the attended talker speak. In fact, participants’ target perception in ‘selective attention’ Experiments 3–5 did not differ from participants who were explicitly instructed to divide their attention equally across the two talkers (Experiment 6). This suggests that contrast effects of speech rate are immune to selective attention, largely operating prior to attentional stream segregation in the auditory processing hierarchy.

## Introduction

Perception typically relies on relative, rather than absolute, coding strategies. That is, perception relies on the encoding of contrast, which enhances processing of information that is most likely to be informative^1,2^. A second key feature of perception is attentional enhancement, which improves the processing of high-priority stimuli in the environment at the expense of less relevant stimuli^3^. These two fundamental processing principles are thought to play a critical role in the ability of biological systems to survive in their typically highly variable environments by allowing them to recognize meaningful items despite variability in their appearance and despite various forms of background noise^4^. Both contrast enhancement and selective attention have been found to operate on a range of perceptual features such as brightness, hue, pitch, loudness, and temperature, to name a few. How they are related to each other is less well understood.

One domain in which these two principles play a critical role is human speech perception. In the case of contrast enhancement, it has been demonstrated that stimulus histories affect the processing of both spectral and temporal information in speech^5–8^. These effects of stimulus history are known as acoustic context effects. To exemplify, when the length of the second unstressed syllable in “terror”/ˈtɛɹ.əɹ/ is gradually decreased, it eventually sounds like the word “tear”/ˈtɛɹ/. Yet, for ambiguous (i.e, perceptually bistable) items, perception of the second syllable is in fact dependent on the rate of surrounding speech. This effect of the contextual speech rate is contrastive: after a fast-spoken sentence an ambiguous token sounds relatively long (compared to the fast sounds of the context), which biases listeners towards perceiving “terror”. Conversely, in the context of a slow sentence, the final syllable sounds relatively short, resulting in the perception of “tear”. That is, in a slow context, syllables can disappear from perception^9,10^. This acoustic context effect induced by the surrounding speech rate, known as a temporal contrast effect or rate normalization, has been shown to influence a wide range of different duration-based phonological cues such as voice onset time (VOT; ^11,12^), formant transition duration^13^, vowel duration^14,15^, lexical stress^16^, and word segmentation^17,18^. In fact, a similar contrastive effect is found in the spectral domain: a sentence with a relatively low first formant (F1) can bias the perception of a following target with an ambiguous F1 (e.g., ambiguous between “bit” and “bet”) towards a high F1 percept (“bet”; known as a spectral contrast effect or spectral normalization^6,19,20^).

Selective attention is also critical in natural spoken communication because it allows listeners to enhance the processing of speech from a specific speaker and/or direction in multi-talker environments (i.e., ‘cocktail party’ settings^21,22^). While most listeners can resolve the multi-talker problem, it poses considerable trouble for automatic speech recognition systems and hearing impaired individuals^23^. One of the ways in which the auditory system deals with competing speech streams is by selectively enhancing the strength of the neural representations of the attended stream in auditory cortex^24,25^, involving a form of gain control^26^. However, little is known about how contrast enhancement and selective attention interact. Recently, evidence has been provided that spectral contrast effects are modulated by selective attention^27^: when presented with two context sentences at the same time, only the spectral properties of the attended sentence influence the perception of a following spectrally ambiguous vowel^28^. However, whether temporal contrast effects are also modulated by selective attention remains unknown.

More specifically, it is unclear whether the speech rate of *unattended* stimuli *does or does not* induce contrastive effects on the perception of a following *attended* target. This issue is highly relevant to speech perception because cocktail parties not only involve different people talking at the same time, those talkers are typically also speaking at different rates. More fundamentally, if unattended speech affects duration perception to the same extent as attended speech, it would support a cognitive processing hierarchy in which temporal contrast effects operate before influences of selective attention^29^. Interestingly, both temporal contrast effects and selective attention affect the processing of sound from early auditory processing levels onwards – but their relative temporal ordering is unknown. For instance, temporal contrast effects modulate the uptake of duration cues immediately upon target presentation and have also been observed for non-speech contexts, such as sequences of tones^5,13^, but see^30^ and even in non-human species^31^. Moreover, acoustic context effects in general also appear when listeners perform demanding concurrent tasks in the visual domain^29^.

One neural mechanism thought to specifically underlie temporal contrast effects involves sustained entrainment of endogenous neural oscillators, phase-locking to the preceding syllabic rate^32^. These entrained neural rhythms have been found to persist for a few cycles after the driving rhythm has ceased^33^, thus influencing the temporal parsing window of following speech segments^5,34,35^. Similarly, the effects of selective attention on auditory perception are thought to occur early in perception^36,37^ and have also been mechanistically explained in terms of phase-locking of low-frequency activity in auditory cortex, but then specifically to the attended speech stream^3,33,34,38^. This overlap in neural mechanisms may hence allow selective gain control to influence temporal contrast effects by enhancing the cortical tracking of attended speech, such that this enhanced entrainment also has a stronger influence on subsequent speech parsing (compared to ignored speech). On the other hand, the sustained influence of neural entrainment that is suggested to underlie temporal contrast effects may in fact originate from earlier (more peripheral) processing stages than the enhancement due to selective attention. Hence, we investigated whether temporal contrast effects reflect the tuning-in to the attended speaker, or whether they precede the influences of attention, being based on the global (combined attended and unattended) sensory environment.

**Figure 1:**
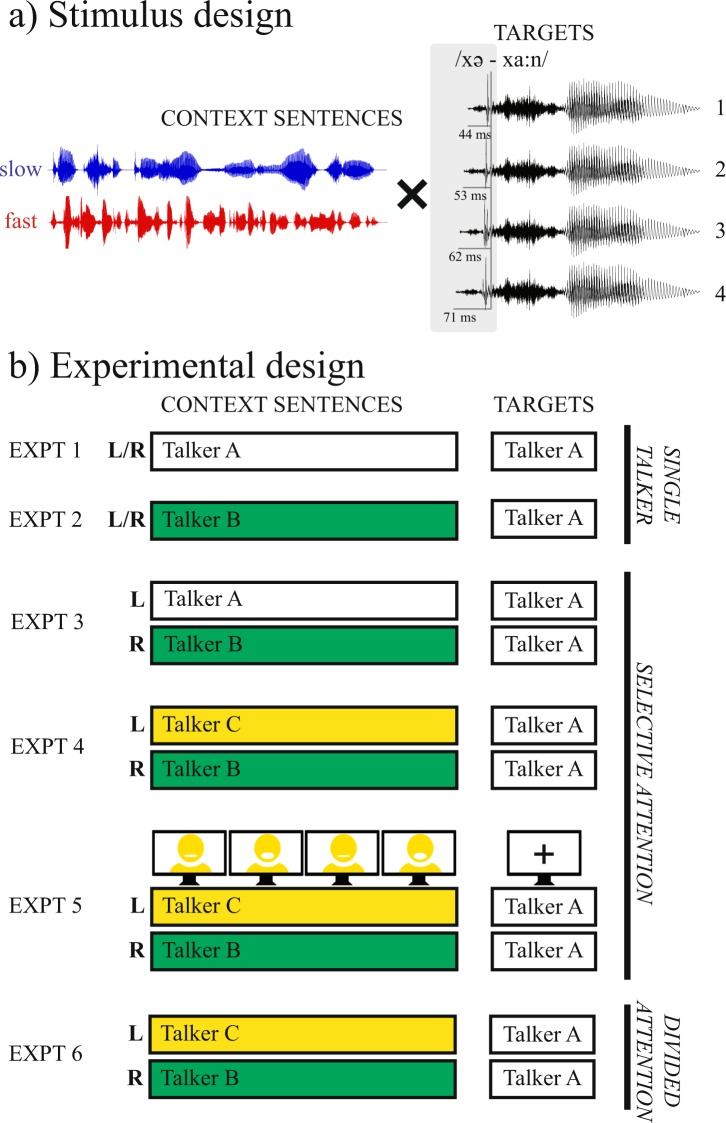
Stimulus design and experimental design of the six experiments. (**a**) Slow and fast context sentences (matched duration) were combined with target sounds, containing an initial syllable /xə-/ with modified durations (e.g., ambiguous between ‘prefix present’ *gegaan* /xə.ˈxa:n/ “gone” and ‘prefix absent’ *gaan* /ˈxa:n/ “to go”). Four-step duration continua of the initial syllable ranged from step 1 (25% of its original duration, most *gaan*-like) to step 4 (40%; most *gegaan*-like). (**b**) Target words were always produced by Talker A (white fill) and preceded by context sentences (with a 100 ms silent gap). Experiments 1 and 2 involved a single talker paradigm, presenting one context sentence at a time (Expt. 1: same talker as target; Expt. 2: different talker as target) at fast and slow rates (on separate, intermixed trials). Experiments 3–5 involved a selective attention paradigm, where participants were instructed to attend one of two different context sentences from two different talkers. In Experiment 3, one context sentence was always produced by Talker A (L/R location counter-balanced across participants) whom participants were instructed to attend. The talker’s speech rate on a given trial varied such that it was fast in one trial, but slow in another trial. The rate of the competing talker could either match or mismatch the rate of the attended talker (both speaking fast or slow vs. one speaking fast, the other slow). Experiment 4 was identical, except that both context sentences were produced by other talkers (B and C). Half of the participants was instructed to attend to Talker B, the other to Talker C. Experiment 5 was identical to Experiment 4 except that a video of the attended talker was provided. Finally, Experiment 6 was identical to Experiment 4, except that it involved a divided attention paradigm: participants were instructed to divide their attention equally across both talkers.

The present study relied on the Dutch morphological prefix *ge-*/xə-/ (e.g., forming the past participle on verbs; e.g., *gegaan* /xə.ˈxa:n/ “gone” vs. *gaan* /ˈxa:n/ “to go”) that is often reduced or elided before /x/-initial stems in spontaneous conversation^39^. We asked Dutch participants to categorize a range of *ge-*initial Dutch words (Supplementary Table S1) that spanned a perceptually ambiguous space between ‘prefix present’ (e.g., *gegaan*) and ‘prefix absent’ forms (*gaan*) by shortening the prefix *ge-* in a number of steps (Fig. 1a). Critically, these target words were preceded by one or two fast (average syllable rate = 5.67 Hz) and slow (2.84 Hz) Dutch context sentences (200 in total; cf. Supplementary Table S2).

In ‘single talker’ Experiments 1–2, we used single context sentences (presented binaurally) followed by target words (Fig. 1b) to demonstrate that slow contexts led participants to miss the initial syllable *ge-* (e.g., more *gaan* reports), even when context sentences were spoken in a different voice than the target. In ‘selective attention’ Experiments 3–5, two lexically different (duration-matched) context sentences were presented to different ears (SNR = 0 dB) with instructions to attend to only one of them. Rate manipulations were fully mixed: on a given trial, one context sentence could be fast or slow, combined with another context sentence being either fast or slow (i.e., intermixed rate-matching and rate-mismatching trials). If selective attention modulated temporal contrast effects induced by preceding speech rates, we would expect a higher proportion of ‘prefix present’ responses when participants attend a fast context sentence, compared to trials in which they attend a slow context sentence, independently of the rate of the competing speaker. If, instead, temporal contrast effects are immune to the influences of selective attention, being based on the global sensory environment, then participants’ target responses in Experiments 3–5 should not differ from participants who are explicitly instructed to divide their attention equally across the two talkers, as in ‘divided attention’ Experiment 6.

## Results

### Slow context sentences make following syllables disappear

Experiments 1 and 2 used a ‘single talker’ paradigm to validate the experimental materials and to serve as a baseline for the following experiments. In particular, they tested whether talker-congruent (Expt. 1) and talker-incongruent (Expt. 2) fast and slow context sentences influence the perceived duration (and hence presence) of the prefix of the target words.

**Figure 2:**
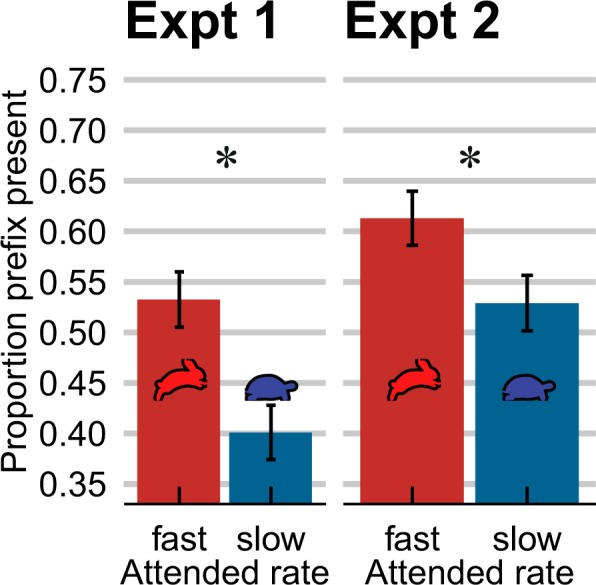
The perception of duration-based speech sound continua is contrastively influenced by the speech rate in the preceding context. In Experiment 1, participants were presented with one context sentence at a time, produced by the same talker as the target. The speech rate of the context sentences had a contrastive effect on target perception: fast context sentences (red bars) led to more ‘prefix present’ responses, slow sentences (blue bars) to more ‘prefix absent’ responses. Changing the talker that produced the context sentences (*talker-incongruent* contexts and targets in Expt. 2) did not change the temporal contrast effect. Error bars enclose 1.96 × SE on either side; 95% confidence intervals. * = p < 0.001.

The categorization results (Fig. 2) demonstrate that slow context sentences resulted in fewer ‘prefix present’ responses than fast sentences, indicating that slow speech rates made the prefix in the target ‘disappear’ (*p* = 0.004), thereby replicating previous findings of temporal contrast effects. Furthermore, the size of the context effect was of a similar size in Experiments 1 and 2 (*p* > 0.05).

### Both attended and unattended contexts influence target categorization

Experiments 3–5 tested whether selective attention could modulate the influence of the context sentences. Participants were presented with two different context sentences, one in each ear, followed by the same target words as in Experiments 1 and 2, spoken by Talker A (cf. Figure 1b). In Experiment 3 participants attended Talker A in one ear and ignored the competing talker in the other ear. In Experiment 4, both context sentences were spoken by voices that differed from the target speaker (to control for the confounding of attention and talker identity that was present in Experiment 3). To aid participants in the focusing of attention, Experiment 5 replicated Experiment 4, but with the addition of a video of the attended talker presented during the context sentence. Across experiments, participants’ success in selective attention was monitored by asking participants to verbally repeat the attended sentence. This was assessed on 20 randomly selected trials (out of the 200; 10%) so participants could not predict when they would be prompted to verbally repeat the attended sentence. They also filled out a post-experimental questionnaire about the perceived difficulty of the attentional task. Verbal repetition scores demonstrated an overwhelming predominance of keywords from the attended talker in ‘selective attention’ Experiments 3–5 (47–64%), indicating successful attention allocation. By comparison, participants in Experiment 6 – who were explicitly instructed to divide their attention equally across the two talkers – were significantly worse at recalling keywords from the sentences (14%; *p* < 0.001).

**Figure 3:**
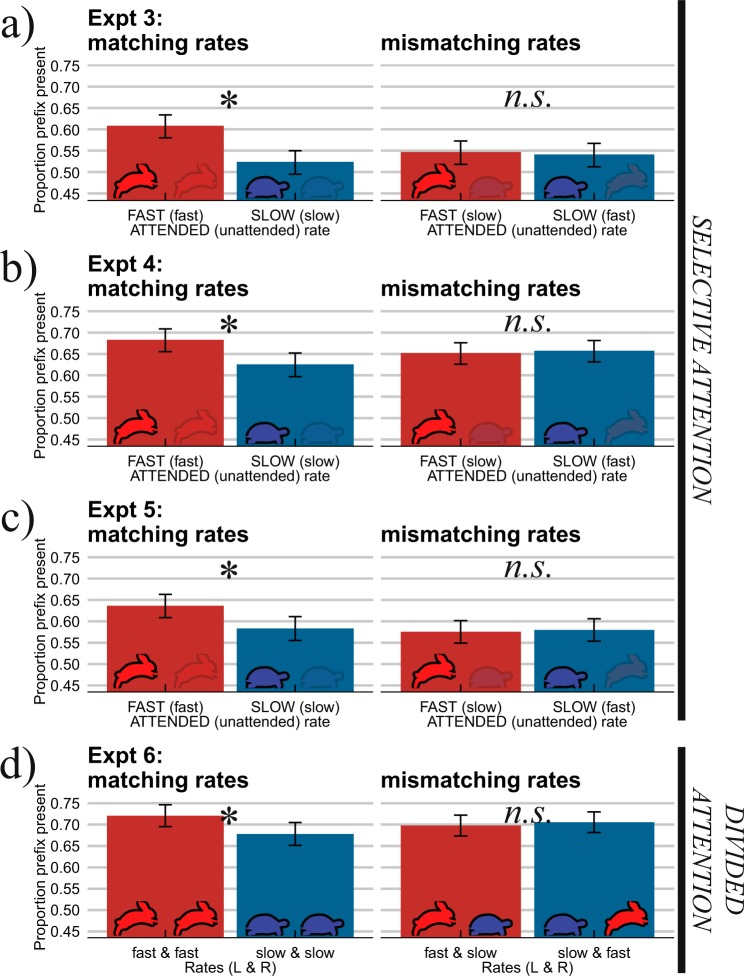
Both attended and unattended contextual speech rates influence target perception to the same degree. When the speech rates of two dichotically presented context sentences match (both fast or slow; left column), fast speech rates bias perception towards ‘prefix present’ responses. However, when the two speech rates mismatch (one slow, the other fast; right column), attending to the fast sentence does not lead to more ‘prefix present’ responses compared to attending to the slow sentence. These results were observed when (**a**) participants attended context sentences in the same voice as the targets (Expt. 3), (**b**) in a different voice than the targets (Expt. 4), and (**c**) even when an additional video of the attended talker was provided (Expt. 5). In fact, participants’ target categorization in ‘selective attention’ Experiments 3–5 did not differ from that of participants in ‘divided attention’ Experiment 6. Attended rates are given in capitals, unattended rates in parentheses. Error bars enclose 1.96 × SE on either side; 95% confidence intervals. * = p < 0.001.

**Figure 4:**
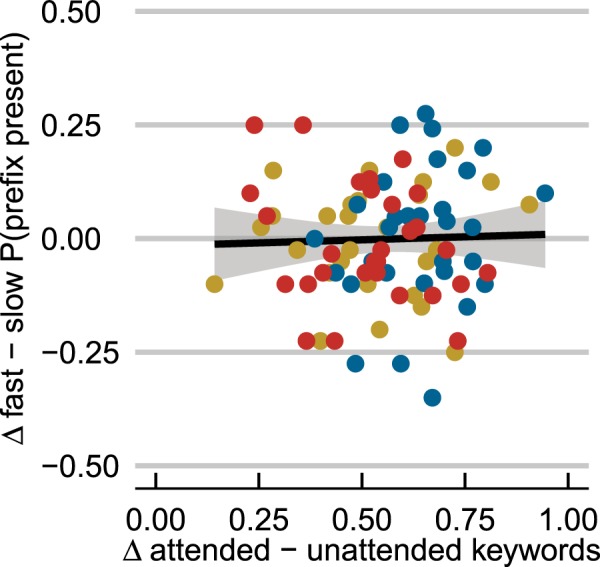
Performance in rate-mismatching conditions is not influenced by participants’ success in selective attention in Experiments 3–5. By-participant variation in selective attention (on x-axis; calculated as difference in proportion keywords correct from attended – unattended context sentence; higher values show greater success) does not predict the difference in proportion ‘prefix present’ responses between the two rate-mismatching conditions (on y-axis; calculated as *P*(prefix present) when attending fast – attending slow) across dichotic Experiments 3–5 (blue = participants from Expt. 3; red = Expt. 4; yellow = Expt. 5): *r* = 0.015; *p* = 0.88.

Results from ‘selective attention’ Experiments 3–5 were very similar (see Fig. 3). That is, no evidence was found, in any experiment, for attentional modulation of temporal contrast effects on target perception. When presented with a fast sentence from one talker and a slow sentence from another talker (i.e., rate-mismatching trials), attending to one or the other sentence did not lead to differential target categorization (no difference between red and blue bars in the right panels in Fig. 3; interaction between Attended Rate and Rate Match: *p* < 0.001; Bayes Factor (*BF*) for Attended Rate in the rate-mismatching conditions = 0.04). Individual variation in how well participants attended the to-be-attended talker also did not predict performance in rate-mismatching conditions (see Fig. 4), suggesting once more that the individual participants’ success at selective attention did not influence target categorization in the two rate-mismatching conditions. In fact, participants’ target categorization behavior in ‘selective attention’ Experiments 3–5 did not differ from that of participants who were explicitly instructed to divide their attention equally across the two talkers (‘divided attention’ Experiment 6). However, across all dichotic Experiments 3–6, a consistent difference was found when both the attended and the unattended sentences had matching speech rates. Two fast context sentences biased perception to ‘prefix present’ responses, while two slow sentences biased towards ‘prefix absent’ responses (*p* < 0.001; *BF* = 208).

## Discussion

Contrast enhancement and selective attention are two well-known processing principles that allow biological systems to break down the substantial variability in their sensory environments, but whether and how the underlying processes interact is poorly understood. We show evidence from temporal contrast effects in speech perception that selective attention does not modulate duration-based contrastive context effects on the perception of speech. Listening to a slow context sentence can make a reduced syllable in a following target word disappear (even when the lead-in sentence is in a different voice than the target; Expts. 1–2), but when the same slow sentence is heard (attended) simultaneously with a competing (ignored) fast talker, this temporal contrast effect is abolished (Expts. 3–5). This observation held for talker-congruent contexts (same voice as targets) and talker-incongruent contexts (different voice than targets). Moreover, the addition of visual articulatory cues to the to-be-attended speech, which is known to considerably aid selective attention in dual-talker environments^40–42^ and to provide additional visual cues to the tempo of the attended talker, did not help reduce the influence of the unattended contexts. In fact, participants’ target categorization in ‘selective attention’ Experiments 3–5 mirrored that of participants in Experiment 6, who were explicitly instructed to divide their attention equally across both talkers.

Duration-based contrastive context effects have been shown in human and non-human perception^31^, by intelligible (speech) and unintelligible (speech/non-speech) contexts^5,13^, but see^30^, and by contexts in a different voice (including one’s own^14,43^). The present study uniquely demonstrates that both attended and unattended contexts equally induce temporal contrast effects, suggesting that rate normalization processes in speech perception are automatic and very general in nature^44^. Moreover, we consistently found that in rate-matching trials hearing two slow context talkers led participants to miss the reduced initial syllable in the targets, while hearing two fast contexts induced more ‘prefix present’ responses. This suggests that temporal contrast enhancement operates over the global sensory environment (independent from selective attentional enhancement), computed over multiple talkers and over longer time periods^45–47^.

We should point out that all participants in the ‘selective attention’ Experiments 3–5 reported to have performed the selective attention task accurately as instructed. This was also evidenced by the high proportion of correct keywords reported from the attended sentences, and the low proportion of keywords reported from the unattended sentences. In fact, the observed proportions of correct keywords are similar to those reported by Bosker, Sjerps, and Reinisch (62–65%^28^), who – using the same paradigm – did find evidence for attentional modulation of *spectral* contrast effects. Moreover, no correlation between individual participants’ success at selective attention and target categorization in rate-mismatching conditions was observed (see Fig. 4). Therefore, the absence of attentional modulation cannot be explained by a presumed failure to accurately attend the to-be-attended talker. It also cannot be explained by participants in Experiments 3–5 purposefully dividing their attention equally across both talkers (i.e., behaving in conflict with the instructions), because divided attention is a very demanding task and leads to lower verbal repetition scores (cf. ‘divided attention’ Experiment 6). Conversely, this also suggests that further facilitation of selective attention, for instance by using two talkers of different gender, is unlikely to provide evidence of attentional modulation (note that the additional visual cues in Experiment 5 also failed to modulate effects).

The observation that selective attention to a particular talker does not modulate the contrast induced by this talker suggests that effects of temporal contrast functionally precede the influence of selective attention. This is in line with the observation that increasing the cognitive demands on attentional resources (cognitive load) also does not result in reduction of acoustic context effects^29^. Still, the fact that temporal contrast enhancement is immune to selective attention is a unique finding. Earlier research indicates that selective attention to one speech stream does not mean that humans can completely ignore unattended sounds. Acoustic and linguistic properties of unattended speech can influence attended speech processing, known as informational masking^48,49^ or attentional leakage^50^. However, in all these cases, the interference from unattended acoustic and linguistic cues is reduced relative to the contribution of attended speech. To our knowledge, this study provides the first demonstration of perceptual processes in speech perception (namely temporal contrast enhancement) that are not modulated by selective attention.

This may be all the more striking considering that both selective attention and temporal contrast effects have been said to involve similar neurobiological mechanisms. Recent MEG^33^ and psychoacoustic^5,34^ evidence suggests that neural oscillators in the theta range (3–9 Hz) become entrained to the fast and slow syllabic rhythms in preceding context sentences. This entrainment is sustained for a few cycles after context sentence offset, influencing how the subsequent target sounds are parsed within the continuous speech stream^5,33^. Solving the ‘cocktail party’ problem in dual-talker listening environments is also said to involve neural representations being selectively phase-locked to the rhythm of each speech stream^51^. Attention modulates the neural representations by enhancing cortical tracking of the attended speech stream^3^. At first sight, this enhanced cortical speech-tracking may predict that following consequences of sustained entrainment should also be modulated by attention. However, selective tracking of the attended talker is most pronounced in higher-order language processing areas and attentional control regions, while the temporal envelope of ignored speech remains robustly represented in lower-level auditory cortex^3,51^. In fact, neural entrainment to the acoustic amplitude fluctuations in speech is comparable in awake (attending) and sleeping (unattending) listeners^52^. The present results advocate a model in which temporal contrast effects are driven by low-level neural entrainment to the syllabic amplitude fluctuations in auditory cortex, unmodulated by attention, which in turn guides subsequent speech parsing.

This study has principally focused on temporal contrast effects (also known as rate normalization), induced by contextual fast and slow speech rates. This raises the question whether all forms of perceptual contrast operate independent from selective attention. Interestingly, recent evidence suggests that spectral contrast effects (i.e., low formant frequencies in context make following formant frequencies sound higher) are modulated by selective attention^27,28^. This indicates that the neurobiological mechanisms underlying these two forms of acoustic context effects are likely to differ (sustained entrained neural oscillators vs. adaptive gain control^20,33,53^). In particular, context effects induced by higher-level properties of language (e.g., based on talker-identity, language, and situation-specific expectations^45,46,54–56^) are likely to be influenced by attention. This advocates a two-stage model of the influence of acoustic context effects^29^, where the earliest perceptual effects are of general auditory nature and unaffected by attention. Additional cognitive effects may emerge at one or more later stages (e.g., decision-making level) during processing, subject to attention allocation.

Beyond fundamental issues, the present study also has practical implications for hearing aid development. The present study has revealed that the syllabic rate of unattended talkers influences the perception of target speech produced by an attended talker. While human listeners may not be able to ignore the temporal envelope of unattended speech, speech enhancement algorithms – as implemented in hearing aids – might. Thus, this study makes reduced transmission of the temporal envelope of unattended talkers a prime target for hearing aid development, potentially aiding attended talker perception in multi-talker settings in hearing impaired individuals.

## Methods

### Participants

Native Dutch individuals with normal hearing were recruited from the Max Planck Institute’s participant pool (Expt. 1: *N* = 16; 11 females (f), 5 males (m); *M*_age_ = 22; Expt. 2: *N* = 16; 13 f, 3 m; *M*_age_ = 24; Expt. 3: *N* = 32; 24 f, 8 m; *M*_age_ = 22; Expt. 4: *N* = 32; 27 f, 5 m; *M*_age_ = 27; Expt. 5: *N* = 32; 28 f, 4 m; *M*_age_ = 23; Expt. 6: *N* = 32; 24 f, 8 m; *M*_age_ = 23). All gave informed consent as approved by the Ethics Committee of the Social Sciences department of Radboud University (project code: ECSW2014-1003-196). All research was performed in accordance with relevant guidelines and regulations. Participants had normal hearing, had no speech or language disorders, and took part in only one of our experiments.

We decided *a priori* to exclude participants with a proportion of ‘prefix present’ responses [*P*(present)] below 0.2 or above 0.8, as for these participants the presented stimuli would be insufficiently ambiguous to establish reliable effects of speech rate. Based on this criterion, 2 additional participants were excluded from Experiment 1 (both < 0.2 *P*(present)), 7 from Experiment 3 (all *P*(present) >0.8); 7 from Experiment 4 (all *P*(present) >0.8); 4 from Experiment 5 (all *P*(present) > 0.8); and 4 from Experiment 6 (all *P*(present) >0.8).

### Stimuli

Two-hundred Dutch context sentences were constructed: half were short (11–13 syllables), the other half long (22–26 syllables; see Supplementary Table S2). All sentences were semantically neutral with regard to the sentence-final target word. Twenty Dutch minimal word pairs were selected as targets. They only differed in the presence vs. absence of the word-initial unstressed syllable /xə-/ (e.g., *gegaan* /xə.ˈxa:n/ “gone” – *gaan* /ˈxa:n/ “to go”; see Supplementary Table S1). This prefix is primarily used to create the past participle, although it can occur in other forms. In spontaneous speech it is often pronounced in a reduced form [x]^39^. If the stem of the word also begins with /x/, the primary difference between the word with and without the prefix is the longer duration of [x]^39^.

Three female native speakers of Dutch (referred to as Talker A, B, and C) were recorded producing all sentences ending in one of the target words. Context sentences (i.e., all speech up to target onset) were excised and manipulated. Each short sentence (11–13 syllables) was paired with one long sentence (22–26 syllables) and their duration was set to the mean duration of that pair across all three talkers using PSOLA in Praat (adjusting tempo while maintaining pitch and formants^57^). That is, the long sentences were compressed and the short sentences were stretched resulting in short sentences played at a slow speech rate and long sentences played at a fast rate, with identical overall duration.

For the target words, only recordings from Talker A were used. Each of the 20 target pairs was manipulated in the initial syllable /xə-/, resulting in duration continua from for instance, ‘prefix absent’ *gaan* to ‘prefix present’ *gegaan* (see Fig. 1a). Four ambiguous steps were created using compression levels of 25%, 30%, 35%, and 40% of the original duration. Two additional unambiguous steps were created for use as filler trials by compressing to 5% (unambiguously *gaan*) and 90% (unambiguously *gegaan*) of the original duration. The intended perception of the duration continuum was confirmed in a pretest presenting manipulated target words in isolation.

### Procedure

In all experiments, participants were presented with combinations of context sentences and target words over headphones (see Fig. 1b). Stimulus presentation was controlled by Presentation software (v16.5; Neurobehavioral Systems, Albany, CA, USA). Each trial started with the presentation of a fixation cross. After 500 ms, the context sentence(s) was presented, followed by a silent interval of 100 ms, followed by a target word. After target offset, the fixation cross was replaced by a screen with two response options (i.e., the words of the minimal pair), one on the left, one on the right (position counter-balanced across participants). Participants entered their response as to which of the two response options they had heard (*gegaan* or *gaan*, etc.) by pressing the “Z” button on a regular computer keyboard for the option on the left, or “M” for the option on the right. After their response (or timeout after 4 seconds), the screen was replaced by an empty screen for 500 ms, after which the next trial was initiated.

Targets were always presented binaurally. They were presented at the four different ambiguous steps of the duration continuum twice: once after a critical slow sentence and once after a critical fast sentence (*N* = 160; henceforth: experimental trials). All target pairs were also presented at the two unambiguous steps of the duration continuum – half following a slow sentence and half following a fast sentence (*N* = 40; henceforth: filler trials).

In Experiment 1, all speech (contexts and targets) was presented binaurally and came from the same talker (Talker A). Experiment 2 was identical, except that the contexts were in a different voice (Talker B or C) than the targets (Talker A). The identity of the context talkers was consistent within but counter-balanced across participants. That is, half listened to context sentences spoken by Talker B, and half to contexts by Talker C.

In Experiment 3, two different context sentences were presented simultaneously to participants, one in each ear (i.e., dichotic presentation; counter-balanced across participants; see Fig. 1b). One of the two context sentences was always produced by the talker that also produced the targets (Talker A; talker-congruent), while the other was produced by a different talker (Talker B or C; talker-incongruent). The identity of the competing talker was consistent within but counter-balanced across participants. Participants were instructed to specifically attend to Talker A throughout the experiment, and ignore the competing talker in the other ear, simulating a ‘cocktail party’ situation. All context sentences were scaled to 70 dB SPL (i.e., 0 dB target-to-masker ratio).

Context sentence and target pairings were based on those from Experiment 1 (all targets presented at all four steps; plus two filler steps), except that during the context, Talker A was only presented in one ear with a competing talker in the other ear. To create rate-matching trials, half of Talker A’s slow sentences was paired with other slow sentences and half of the fast sentences was paired with other fast sentences, each from the other talkers, of different semantic content, but with the same number of syllables. To create rate-mismatching sentences fast and slow sentences were paired, using the pairs described for the rate manipulation above.

For Experiment 4, the same context pairs and target combinations were used as in Experiment 3, except that this time the context by Talker A was replaced by the same sentence from another talker (Talker B or C). Participants in Experiment 4 were randomly allocated to one of two groups. One group (*n* = 16) was instructed to selectively attend to Talker B (and ignore Talker C), while the other group (*n* = 16) was instructed to focus on Talker C (and ignore Talker B). Thus, each group of participants listened to the exact same acoustic stimuli; they only differed in which talker they attended to. The location of the attended talker (i.e., which ear) was counter-balanced across participants. This meant that participants selectively attended to only one ear + talker combination throughout the experiment. Context Rate was varied within participants on a trial-by-trial basis; that is, in one trial the attended talker spoke fast, on another the attended talker spoke slowly, etc.

Experiment 5 was similar to Experiment 4, except that an additional video of the attended talker was presented during the context window (video dimensions: 960 by 580 pixels). Also, because we had only recorded audio (no video) for the previous experiments, we re-recorded the context sentences used in the previous experiments. Two new female native speakers of Dutch were video-recorded (‘talking head’ format) while reading out all context sentences ending in one of the target words. All stimulus manipulations, context pairings, and target combinations were similar to Experiment 4, except that these were now performed using *atempo* in *FFmpeg* (open source multimedia software; http://www.ffmpeg.org).

Finally, Experiment 6 followed the same procedure as Experiment 4, except that participants were explicitly instructed to *divide their attention* across both talkers at the same time.

### Verbal repetition

In order to verify that participants in ‘selective attention’ Experiments 3–5 were indeed selectively attending to one talker and ignoring the other, participants were presented with prompts to type out the last attended sentence. These prompts were presented after half of the filler trials (*n* = 20 out of 200 trials) after they had provided a categorization response. Because trials were randomized within participants, participants could not predict on which trials they would be prompted to repeat the attended sentence. Additionally, after the experiment, they were asked to fill out a debriefing questionnaire about the perceived difficulty of the attentional task, how successful they were in this task, and potential strategies.

The mean proportion of keywords reported from the *attended/unattended* sentences were: Experiment 3, 0.64/0.03 (*SD* = 0.31/0.13); Experiment 4, 0.48/0.06 (*SD* = 0.33/0.18); Experiment 5, 0.47/0.08 (*SD* = 0.33/0.22). In Experiment 3, participants reported more keywords from the unattended than the attended sentence in only 4% of the prompts (Expt. 4: 10%; Expt. 5: 13%). These percentages were comparable in trials with matching and mismatching rates.

In ‘divided attention’ Experiment 6, verbal repetition prompts were also presented after a randomly selected 10% of the trials. However, in Experiment 6, participants were asked to verbally repeat one of the two sentences they had divided their attention across (left vs. right sentence was selected randomly for each trial). Hence, participants could not predict when or which ear they would be prompted to verbally repeat, further motivating them to divide their attention equally across the two talkers. The mean proportion of keywords reported from the prompted sentences in Experiment 6 was only 0.14 (*SD* = 0. 23). A Linear Mixed Model^58^ on the logit-transformed proportion data revealed that performance in Experiment 6 was significantly lower than all three selective attention experiments (Expt. 6 vs. 3: *β* = 3.428, *SE* = 0.148, *t* = 23.150, *p* < 0.001; Expt. 6 vs. 4: *β* = 2.125, *SE* = 0.146, *t* = 14.580, *p* < 0.001; Expt. 6 vs. 5: *β* = 2.150, *SE* = 0.140, *t* = 15.420, *p* < 0.001). In sum, while divided attention was a difficult task, participants in Experiments 3–5 were largely successful at selectively attending to one talker while ignoring the other competing talker.

This was further corroborated by participants’ responses on the questionnaire. These indicated that the selective attention task was demanding but doable. At times, the attentional focus was lost but most participants reported to restore selective attention by, for instance, looking in the direction of the attended sound, concentrating carefully, or silently shadowing the attended talker. It seemed that participants in Experiment 5 found the selective attention task easier, likely due to the additional visual cues to the to-be-attended speech stream. In contrast, all participants from Experiment 6 reported that dividing their attention across the two talkers equally was very difficult. They frequently failed to divide their attention, instead attending one talker on some trials, and the other talker on other trials. Several participants reported the speech rate manipulation.

### Statistical analysis

Trials with missing categorization responses due to timeout (Expt. 1: *n* = 9; <1%; Expt. 2: *n* = 16; <1%; Expt. 3: *n* = 44; 1%; Expt. 4: *n* = 9; <1%; Expt. 5: *n* = 5; <1%; Expt. 6: *n* = 16; <1%) were excluded from analyses. The binomial categorization data in experimental trials were analyzed using Generalized Linear Mixed Models (GLMM^59^) with a logistic linking function as implemented in the lme4 library (version 1.0.5^60^) in R^61^. The binomial dependent variable was participants’ categorization of the target as either the ‘prefix present’ (e.g., *gegaan*; coded as 1) or the ‘prefix absent’ target word (e.g., *gaan*; coded 0). Analyses were run separately for the ‘single talker’ Experiments 1–2 and the dichotic Experiments 3–6.

The data from the ‘single talker’ Experiments 1–2 were combined and analyzed for fixed effects of Continuum Step (continuous predictor; centered and scaled around the mean), Context Rate (categorical predictor; deviation coding, with slow context coded as −0.5 and fast as +0.5), Experiment (categorical predictor; dummy coding, with Experiment 2 mapped onto the intercept), and all interactions. The model included Participant and Target Pair as random factors, with by-participant and by-item random slopes for Context Rate. Models with more complex random effects structures failed to converge. This model revealed significant effects of Continuum Step (*β* = 1.539, *SE* = 0.065, *z* = 23.540, *p* < 0.001; higher *P*(present) as initial syllable duration increased) and Context Rate (*β* = 0.627, *SE* = 0.218, *z* = 2.874, *p* = 0.004; higher *P*(present) for contexts with fast speech rates). We also found a main effect of Experiment (*β* = −0.775, *SE* = 0.378, *z* = −2.052, *p* = 0.040), revealing that there was a significantly higher proportion of ‘prefix present’ responses in Experiment 2 compared to Experiment 1 (*M*_*Expt1*_ = 0.47; *M*_*Expt2*_ = 0.57). No interactions were observed, suggesting that the effect of Context Rate did not differ across these two single talker experiments.

The data from the four dichotic Experiments 3–6 were analyzed for fixed effects of Continuum Step (continuous predictor; centered and scaled around the mean), Attended Rate (categorical predictor; deviation coding, with slow attended rate coded as −0.5 and fast attended rate as +0.5), Rate Match (categorical predictor; deviation coding, with rate-mismatching trials coded as −0.5 and rate-matching trials as +0.5), Experiment (categorical predictor; dummy coding, with Experiment 3 mapped onto the intercept), and all interactions. The model included Participant and Target Pair as random factors, with by-participant and by-item random slopes for Context Rate. Models with more complex random effects structures failed to converge. This model revealed significant effects of Continuum Step (*β* = 1.527, *SE* = 0.045, *z* = 33.751, *p* < 0.001; higher *P*(present) as initial syllable duration increased) and an effect of Attended Rate (*β* = 0.341, *SE* = 0.091, *z* = 3.742, *p* < 0.001; higher *P*(present) when attending a fast vs. slow context sentence). However, there was an interaction between Attended Rate and Rate Match (*β* = 0.599, *SE* = 0.155, *z* = 3.866, *p* < 0.001), suggesting a difference between the two rate-matching conditions but no difference between rate-mismatching conditions. In fact, no 3-way interaction between Attended Rate, Rate Match, and Experiment was observed, indicating similar categorization behavior across all four dichotic experiments – regardless of whether they involved a ‘selective attention’ (Experiments 3–5) or a ‘divided attention’ paradigm (Experiment 6). Separate analyses of rate-matching and rate-mismatching trials revealed only an effect of Attended Rate in rate-matching trials (*β* = 0.649, *SE* = 0.141, *z* = 4.602, *p* < 0.001; higher *P*(present) for two fast than two slow contexts), but no effect in rate-mismatching trials (*β* = 0.039, *SE* = 0.145, *z* = 0.269, *p* = 0.788).

We also found an overall difference between Experiment 3 and 4 (*β* = 0.660, *SE* = 0.313, *z* = 2.111, *p* = 0.035) and Experiment 3 and 6 (*β* = 1.025, *SE* = 0.314, *z* = 3.268, *p* = 0.001), revealing that there was a significantly higher proportion of ‘prefix present’ responses in Experiment 4 and Experiment 6 compared to Experiment 3 (*M*_*Expt3*_ = 0.56; *M*_*Expt4*_ = 0.65; *M*_*Expt3*_ = 0.59; *M*_*Expt4*_ = 0.70). An overall effect of Rate Match (*β* = 0.185, *SE* = 0.077, *z* = 2.387, *p* = 0.017) indicated a slightly higher proportion of ‘prefix present’ responses in rate-matching (*P*(present) = 0.63) vs. rate-mismatching trials (*P*(present) = 0.62). Finally, there was a small interaction between Continuum Step and the contrast between Experiment 3 and 4 (*β* = −0.150, *SE* = 0.061, *z* = −2.450, *p* = 0.014) and between Continuum Step and the contrast between Experiment 3 and 6 (*β* = −0.212, *SE* = 0.063, *z* = −3.343, *p* < 0.001), suggesting a slightly reduced effect of Continuum Step in Experiment 4 and 6 compared to Experiment 3.

Finally, Bayesian analyses were carried out (see Supplementary Information) corroborating, first, the absence of evidence for a difference between the two rate-mismatching conditions; second, strong evidence for the alternative hypothesis that the two rate-matching conditions do differ in dichotic Experiments 3–6.

## Supplementary information

Supplementary Information.

## Data Availability

The datasets generated and analyzed during the current study are available in the Open Science Framework repository: https://osf.io/dp7ck.
