# Supplementary material for: Temporal contrast effects in human speech perception are immune to selective attention
Source: Sci Rep. 2020 Mar 27;10:5607. doi: 10.1038/s41598-020-62613-8 (PMC7101381; doi:10.1038/s41598-020-62613-8)
Supplement: Supplementary file 1 — Supplementary Information. [file 41598_2020_62613_MOESM1_ESM.docx]

Supplementary Information

Temporal contrast effects in human speech perception are immune to selective attention

Hans Rutger Bosker, Matthias Sjerps, and Eva Reinisch

Corresponding author: Hans Rutger Bosker

Email: [HansRutger.Bosker@mpi.nl](mailto:HansRutger.Bosker@mpi.nl)

**This Supplementary Information file includes:**

Supplementary text

Bayesian analyses

Supplementary Tables S1-S2

References for Supplementary Information citations

Supplementary Information Text

**Bayesian analyses.** The absence of evidence for a difference between the two rate-mismatching conditions was further corroborated by a Bayesian analysis (using the package BayesFactor in R; version 0.9.11-1) ^1^. Because there is as yet no Bayesian analogue of linear mixed effects models, we ran a Bayesian linear regression. First, we calculated the average *P*(present) for each participant from Experiments 3-6, separately for each continuum step, for each of the four Attended Rate conditions. After logit transformation (average proportions 0 and 1 were avoided by adding or subtracting 0.01 to circumvent infinity values), these data were entered into Bayesian linear models, separately for rate-matching and rate-mismatching trials. For rate-mismatching trials, the numerator model involved the fixed factors Experiment, Continuum Step, and Attended Rate, with random intercepts for Participant. The denominator model was identical except that it did not include the predictor Attended Rate. As such, the comparison of the numerator and denominator model tests the contribution of Attended Rate, with Bayes Factors below 1/3 providing evidence for the null hypothesis ^2^. The Bayes Factor (*BF*) for the Attended Rate predictor was .04, hence demonstrating that there was no difference between the two rate-mismatching conditions. However, a similar comparison for rate-matching trials revealed *BF* = 208, providing strong evidence for the alternative hypothesis that the two rate-matching conditions do differ.

Supplementary Table S1

List of target minimal pairs (in *italics*; *N* = 20) with IPA transcriptions and English translations.

|  | ‘prefix absent’ member | ‘prefix present’ member |
| --- | --- | --- |
| 1 | *gaan* /'xa:n/ “to go” | *gegaan* /xə.'xa:n/ “gone” |
| 2 | *geven* /'xe.və/ “to give” | *gegeven* /xə.'xe.və/ “given” |
| 3 | *garandeert* /'xa:.rɑn.de:rt/ “guarantees” | *gegarandeerd* /xə.'xa:.rɑn.de:rt/ “guaranteed” |
| 4 | *gapt* /'xɑpt/ “steals” | *gegapt* /xə.'xɑpt/ “stolen” |
| 5 | *grift* /'xrɪft/ “inscribes” | *gegrift* /xə.'xrɪft/ “inscribed” |
| 6 | *gaart* /'xa:rt/ “cooks” | *gegaard* /xə.'xa:rt/ “cooked” |
| 7 | *gaapt* /'xa:pt/ “yawns” | *gegaapt* /xə.'xa:pt/ “yawned” |
| 8 | *galoppeert* /xɑ.lɔ.'pe:rt/ “gallops” | *gegaloppeerd* /xə.xɑ.lɔ.'pe:rt/ “galloped” |
| 9 | *glazuurt* /xla:.'zy:rt/ “glazes” | *geglazuurd* /xə.xla:.'zy:rt / “glazed” |
| 10 | *gijzelt* /'xɛi.zəlt/ “takes hostage” | *gegijzeld* /xə.'xɛi.zəlt/ “taken hostage” |
| 11 | *graast* /'xra:st/ “grazes” | *gegraasd* /xə.'xra:st/ “grazed” |
| 12 | *golft* /'xɔlft/ “plays golf” | *gegolft* /xə.'xɔlft/ “played golf” |
| 13 | *geeuw* /'xe:u/ “yawn” | *gegeeuw* /xə.'xe:u/ “yawning” |
| 14 | *gil* /'xɪl/ “scream” | *gegil* /xə.'xɪl/ “screaming” |
| 15 | *gniffel* /'xnɪ.fəl/ “chuckle” | *gegniffel* /xə.'xnɪ.fəl/ “chuckling” |
| 16 | *giechel* /'xi.xəl/ “giggle” | *gegiechel* /xə.'xi.xəl/ “giggling” |
| 17 | *grom* /'xrɔm/ “growl” | *gegrom* /xə.'xrɔm/ “growling” |
| 18 | *goed* /'xut/ “good” | *gegoed* /xə.'xut/ “wealthy” |
| 19 | *gier* /'xir/ “vulture” | *gegier* /xə.'xir/ “shrieking” |
| 20 | *galm* /'xɑlm/ “reverberation” | *gegalm* /xə.'xɑlm/ “reverberation” |

Supplementary Table S2

List of context sentences (*N* = 200) with number of syllables (σ) and English paraphrases.

|  | Dutch context sentence | σ | English (literal) paraphrase |
| --- | --- | --- | --- |
| 1 | In het tijdschrift stonden twee keer strepen bij het woordje | 11 | In the magazine stood two stripes near the word |
| 2 | Ellen hoort de dikwijls stomme moppen met het woordje | 11 | Ellen hears often stupid jokes with the word |
| 3 | Slimme dokters kopen dikke boeken met het woordje | 11 | Smart doctors buy thick books with the word |
| 4 | Zeven grote kerels spelen spellen met het woordje | 11 | Seven tall guys play games with the word |
| 5 | Deze lieve moeder stuurde foto's met het woordje | 11 | This sweet mother sent pictures with the word |
| 6 | Stille dichters horen vele spreuken met het woordje | 11 | Silent poets hear many sayings with the word |
| 7 | Jonge mensen schrijven mooie brieven met het woordje | 11 | Young people write beautiful letters with the word |
| 8 | Jonge kerels lezen boekjes met hierin het woordje | 11 | Young guys read little books with the word |
| 9 | Coole DJ's spelen korte stukken met het woordje | 11 | Cool DJ's play short fragments with the word |
| 10 | Rijke jongens horen droge moppen met het woordje | 11 | Rich boys hear dry jokes with the word |
| 11 | Deze luie schrijvers roepen zinnen met het woordje | 11 | These lazy writers yell sentences with the word |
| 12 | Deze kleine kindjes krijgen lessen met het woordje | 11 | These little children receive lessons with the word |
| 13 | Slimme mensen zetten soms een lidwoord voor het woordje | 11 | Smart people put sometimes an article before the word |
| 14 | Deze snelle fietsers moeten stoppen bij het woordje | 11 | These fast cyclists must stop near the word |
| 15 | Deze grote schermen tonen errors in het woordje | 11 | These large screens show errors in the word |
| 16 | Hoge torens zenden nieuwtjes met hierin het woordje | 11 | High towers send news with in it the word |
| 17 | Deze enge beesten schrikken telkens ernstig door het woordje | 11 | These scary beasts are scared heavily by the word |
| 18 | Tommy tilde zeven volle koffers met erop het woordje | 11 | Tommy lifted seven full suitcases with on them the word |
| 19 | 's Morgens zongen deze meisjes liedjes met het woordje | 11 | In the morning the girls sung songs with the word |
| 20 | Willem loerde heen en weer en riep toen plots het woordje | 11 | Willem lurked back and forth and yelled suddenly the word |
| 21 | Boven deze rood geverfde schutting klonk het woordje | 11 | Above the red painted fence sounded the word |
| 22 | Kindjes zingen met plezier het versje met het woordje | 11 | Little children sing with pleasure the verse with the word |
| 23 | Toen de keuken opgeruimd werd zei hij plots het woordje | 11 | When the kitchen cleaned was said he suddenly the word |
| 24 | Op de banner voor de grote voordeur stond het woordje | 11 | On the banner in front of the large front door stood the word |
| 25 | Dolly smeekte Kitty mee te denken voor het woordje | 11 | Dolly begged Kitty with to think for the word |
| 26 | Onno kon zich moeilijk concentreren op het woordje | 11 | Onno could himself hardly concentrate on the word |
| 27 | Moeder kon geeneens een zin bedenken met het woordje | 11 | Mother could not even a sentence think with the word |
| 28 | Tinus kreeg geschreven brieven met erop het woordje | 11 | Tinus received written letters with on them the word |
| 29 | Ieder mens vergeet wel eens te kiezen voor het woordje | 11 | Every human forgets sometimes to choose for the word |
| 30 | Deze winkeliers vertoonden grote zorgen om het woordje | 11 | These shopkeepers showed great concern about the word |
| 31 | Mirthe vond de roze koekjes lijken op het woordje | 11 | Mirthe thought the pink biscuits looked like the word |
| 32 | Onze broertjes schrijven zotte spreuken met het woordje | 11 | Our little brother wrote foolish sayings with the word |
| 33 | Grote groene bomen zijn beschreven met het woordje | 11 | Tall green trees are described with the word |
| 34 | Volgens deze teksten moet je letten op het woordje | 11 | According to these texts must you pay attention to the word |
| 35 | Kleine peuters geven doosjes met erin het woordje | 11 | Little toddlers give boxes with in them the word |
| 36 | Timo kon zijn liefde niet bedwingen voor het woordje | 11 | Timo could his love not contain for the word |
| 37 | Lotte moest voor hem een boekje kopen met het woordje | 11 | Lotte had to for him a booklet buy with the word |
| 38 | Hij verzond voor hem een mooie foto met het woordje | 11 | He sent for him a beautiful picture with the word |
| 39 | Op het beeldscherm knippert nog een cursor bij het woordje | 11 | On the screen blinked still a cursor near the word |
| 40 | Peter wilde in zijn teksten kiezen voor het woordje | 11 | Peter wanted in his texts to choose for the word |
| 41 | Ilse vroeg of Simon nog een zin wist met het woordje | 11 | Ilse asked if Simon another a sentence knew with the word |
| 42 | Midden door de speech die moeder voordroeg riep zij het woordje | 12 | Middle through the speech that mother recited yelled she the word |
| 43 | Deze kleine blonde meisjes leren met hulp het woordje | 12 | These little blonde girls learn with help the word |
| 44 | In het proefwerk over spelling kiest hij weer voor het woordje | 12 | In the test about spelling chooses he again for the word |
| 45 | Zij heeft moeite met de spelling in de zin met het woordje | 12 | She has trouble with spelling in the sentence with the word |
| 46 | Dit kleine kindje zingt duistere liedjes met het woordje | 12 | This little child sings dark songs with the word |
| 47 | Leuke meiden verzinnen diepe teksten met het woordje | 12 | Nice girls make up deep texts with the word |
| 48 | Trotse meisjes lezen tijdschriften met hierin het woordje | 12 | Proud girls read magazines with in them the word |
| 49 | Dunne jongetjes krijgen folders met hierop het woordje | 12 | Slender little boys receive flyers with on them the word |
| 50 | Dikwijls hebben veel mensen grote ruzie om het woordje | 12 | Often have many people big fights about the word |
| 51 | Deze bekende boekjes hebben titels met het woordje | 12 | These well-known booklets have titles with the word |
| 52 | In de lezing zei de juf wijze dingen met het woordje | 12 | In the lecture the teacher said wise things with the word |
| 53 | Deze kindjes kopen potloden met hierop het woordje | 12 | The little children buy pencils with on it the word |
| 54 | Deze eerste zin die ze opschreef begon met het woordje | 12 | This first sentence that she wrote started with the word |
| 55 | Deze meisjes springen op leuke liedjes met het woordje | 12 | These girls jump to the nice songs with the word |
| 56 | Op het witte bureau liggen vijf brieven met het woordje | 12 | On the desk are five letters with the word |
| 57 | In de vriezer ligt een doos ijsjes met erop het woordje | 12 | In the freezer lies a box popsicles with on them the word |
| 58 | In de felle zon weerspiegelt de schone ruit het woordje | 12 | In the bright sun reflects the clean window the word |
| 59 | Op de lichtgekleurde muur stond er in het rood het woordje | 12 | On the light-colored wall stood there in the red the word |
| 60 | Deze strenge meesters schrijven op het schoolbord het woordje | 12 | The strict teachers write on the blackboard the word |
| 61 | Toen bestelde hij negen rode rozen met het woordje | 12 | Then he ordered nine red roses with the word |
| 62 | Blije kindjes lopen door de steeg en roepen het woordje | 12 | Happy little children walk through the alley and yell the word |
| 63 | Deze sluwe jongen fluistert opnieuw discreet het woordje | 12 | This cunning boy whispers again discretely the word |
| 64 | Tijdens de vliegreis richting Londen typte hij het woordje | 12 | During the flight to London typed he the word |
| 65 | Merel sloeg het schrift open in de wind en schreef het woordje | 12 | Merel slammed the notebook open in the wind and wrote the word |
| 66 | 's Ochtends liep hij door het huis en vond de brief met het woordje | 12 | In the morning walked he through the house and found the letter with the word |
| 67 | Toen hij deze brieven schreef kreeg hij twijfels bij het woordje | 12 | When he these letters wrote got he doubt at the word |
| 68 | Deze blonde meiden zijn heel tevreden met het woordje | 12 | These blonde girls are very satisfied with the word |
| 69 | Zij probeert poezie te construeren met het woordje | 12 | She tries poetry to construct with the word |
| 70 | Deze onbekende schrijver schreef een boek met het woordje | 12 | This unknown writer wrote a book containing the word |
| 71 | Bettie ging ons tuinhok in en schreef op de muur het woordje | 12 | Bettie went our garden shed in and wrote on the wall the word |
| 72 | Onze mooie witte shirts zijn beschreven met het woordje | 12 | Our beautiful white shirts are bewritten with the word |
| 73 | Deze nieuwe enquetes zijn beschreven met het woordje | 12 | These new surveys are described with the word |
| 74 | Hij is bezig rijmwoorden voor te stellen voor het woordje | 12 | He is busy rhyming words to propose for the word |
| 75 | De jongen wilde niet zijn huiswerk doen en riep het woordje | 12 | The boy want not his homework do and yelled the word |
| 76 | Tijdens het congres benoemt zij problemen met het woordje | 12 | During the congress names she problems with the word |
| 77 | Jos kleurde mooie tekeningen met erbij het woordje | 12 | Jos colored beautiful drawings with with them the word |
| 78 | Deze tiener vormde herinneringen bij het woordje | 12 | This teenager formed memories at the word |
| 79 | Mensen die hem kenden steunden zijn gevoel bij het woordje | 12 | People that him knew supported his feeling ... the word |
| 80 | Tony en zijn vrienden toonden geen moeite met het woordje | 12 | Tony and his friends showed no trouble with the word |
| 81 | Zij probeerden deze ruzie op te lossen met het woordje | 13 | They tried this argument to solve with the word |
| 82 | Deze postdoc moest zijn proefschrift controleren op het woordje | 13 | This postdoc had to his dissertation check for the word |
| 83 | Deze ronde keien op de vloeren lijken op het woordje | 13 | These round cobblestones on the floors look like the word |
| 84 | Bij het lezen in het boekje stopte Dennis bij het woordje | 13 | With the reading in the booklet stopped Dennis at the word |
| 85 | Tijdens onze meeting zette hij een stipje bij het woordje | 13 | During our meeting put he a dot at the word |
| 86 | De leerling begint elke zin in zijn werkstuk met het woordje | 13 | The student starts every sentence in his paper with the word |
| 87 | Deze mensen hebben grote vellen met hierop het woordje | 13 | These people have large sheets with on them the word |
| 88 | Studenten eten brood uit een trommel met erop het woordje | 13 | Students eat bread from a lunch box with on it the word |
| 89 | Vele mensen snoeien heggen bij de woning met het woordje | 13 | Many people trim hedges near the house with the word |
| 90 | Het kind koopt snoep bij de winkel in de speeltuin met het woordje | 13 | The child buys candy at the store in the playground with the word |
| 91 | Zeven jongens zitten buiten op het stoepje bij het woordje | 13 | Seven boys sit outside on the step near the word |
| 92 | Onze honden hebben leuke speeltjes met erop het woordje | 13 | Our dogs have nice toys with on them the word |
| 93 | Uit de bomen vielen roze bloemen met erop het woordje | 13 | From the trees fell pink flowers with on them the word |
| 94 | Kindjes uit de zwemles gingen 's ochtends spelen bij het woordje | 13 | Children from the swimming lesson went in the morning to play at the word |
| 95 | Tijdens dit snelle spel verloren veel mensen met het woordje | 13 | During this quick game lost many people with the word |
| 96 | Tijdens deze conferentie moest hij gieren om het woordje | 13 | During this conference had to he shriek at the word |
| 97 | Gertje is zeven weken luidruchtig bezig met het woordje | 13 | Gertje is seven weeks loudly busy with the word |
| 98 | Op het scherm op zijn mobiel stond een tekst met erin het woordje | 13 | On the screen on his mobile stood a text with in it the word |
| 99 | Toen zij richting deze winkel liepen zeiden zij het woordje | 13 | When she direction this store walked said they the word |
| 100 | De coach uit het roeiteam liep in een broek met erop het woordje | 13 | The coach from the rowing team walked in pants with on them the word |
| 101 | Toen de strenge meester ontdekte wie de school te vroeg verliet, riep hij met luide stem het woordje | 22 | When the strict teacher discovered who the school too early left, yelled he with loud voice the word |
| 102 | Tot zijn grote ongenoegen struikelde hij tijdens deze spellingswedstrijd over het woordje | 22 | To his great dissatisfaction stumbled he during this spelling competition over the word |
| 103 | De verzorger liep met twee kilo mest in het bos richting zijn koeien en hoorde toen het woordje | 22 | The caretaker walked with two kilos of dung in the forest towards his cows and heard then the word |
| 104 | Tijdens de eerste toets op zijn nieuwe school lukte het hem niet om te concentreren op het woordje | 22 | During the first test at his new school manage it him not to concentrate on the word |
| 105 | Voor het uitgebreide ontbijt fluisterde zijn ontdeugende nichtje hem in zijn oor het woordje | 22 | Before the elaborate breakfast whispered his naughty cousin him in his ear the word |
| 106 | Sommige mensen op vreemde plekken gebruiken in een gekke bui heel geregeld het woordje | 22 | Some people in strange places use in a silly mood very regularly the word |
| 107 | Sommige meisjes kunnen heel veel kletsen over kleding en spreken uren over het woordje | 22 | Some girls can a lot chat about clothing and speak hours about the word |
| 108 | De blonde meid wilde het liefst een ontzettend griezelig boek lezen met in de titel het woordje | 22 | The blonde girl wanted the most an awfully scary book read with in the title the word |
| 109 | Toen er een code nodig bleek te zijn moest iedereen een term bedenken lijkend op het woordje | 22 | When there a code needed turned out to be had to everyone a term think of similar to the word |
| 110 | Volgens de jonge kinderen is tijdens het grote dictee het moeilijkste woordje het woordje | 22 | According to the young children is during the great dictation the most difficult word the word |
| 111 | Toen de reizigers nieuws zochten over het treinverkeer, verscheen er op het grote bord het woordje | 22 | When the travelers news looked for about the railway traffic, appeared there on the large sign the word |
| 112 | Tijdens het spelletje pictionary lukte het de jongen niet te komen op het woordje | 22 | During the game pictionary, succeed it the boy not to think of the word |
| 113 | Op de sticker die de blije meisjes bij hun ijsjes kregen stond heel groot geschreven het woordje | 22 | On the sticker that the happy girls with their ice cream got, was very large written the word |
| 114 | Op het veldje met de grote bomen stond een verstrooide zwerfer die iets riep over het woordje | 22 | On the field with the big trees stood an absent-minded hobo/tramp, who shouted something about the word |
| 115 | Toen de juf de koptelefoon op zette hoorde zij veel bekende woorden en ook het woordje | 22 | When the teacher the headphones put on heard she many well-known words and also the word |
| 116 | Toen de zorgvuldig neergezette dominostenen omvielen, schreeuwde ze woedend het woordje | 22 | When de carefully placed dominos fell over, yelled she furious the word |
| 117 | Gisterochtend tijdens het ontbijt werd moeder gebeld en voerde een gesprek over het woordje | 22 | Yesterday morning during the breakfast was mother called and had a conversation about the word |
| 118 | De omringende mensen konden hun ogen niet geloven toen het meisje wees op het woordje | 22 | The surrounding people could their eyes not believe when the girl pointed out the word |
| 119 | De twee vriendinnen bleven voor de korte voorstelling de hele tijd gieren over het woordje | 22 | The two girlfriends kept before the short show the whole time shrieking about the word |
| 120 | In de concertruimte zongen de tenoren in koor een geweldig lied met erin het woordje | 22 | In the concert room sang the tenors in chorus an amazing song with in it the word |
| 121 | De schrijfster vond het nog steeds ontzettend moeilijk om een tekst te verzinnen met erin het woordje | 22 | The writer found it still very difficult to a text make up with in it the word |
| 122 | Geheel onbedoeld spelden de vermicelliletters in zijn grootmoeder's groentesoep het woordje | 22 | Completely unintentionally spelled the vermicelli letters in his grandmothers vegetable soup the word |
| 123 | De kinderboekenschrijver vond het heel moeilijk om synoniemen te bedenken voor het woordje | 22 | The children's books author found it very difficult to synonyms to make up for the word |
| 124 | Hij hoorde de groep vriendinnen in de drogisterij de hele tijd kletsen over het woordje | 22 | He heard the group of girlfriends in the drugstore the whole time chatting about the word |
| 125 | Toen het beroemde jurylid het modetijdschrift open sloeg, viel zijn oog meteen op het woordje | 22 | When the famous juror the fashion magazine opened (out), fell his eye immediately on the word/saw he immediately the word |
| 126 | Toen de klinisch psycholoog hem vroeg om zijn dromen op te schrijven, schreef hij in het schrift het woordje | 22 | When the clinical psychologist him asked to his dreams to write down, wrote he in the notebook the word |
| 127 | De stoere motorrijder wilde stiekem toch een roze helm kopen met bovenop het woordje | 22 | The tough motorcyclist wanted secretly still a pink helmet buy with on top the word |
| 128 | Op het hoge podium stond de spreker breed te grijnzen bij de opmerking over het woordje | 22 | On the high stage stood the speaker widely grinning at the remark about the word |
| 129 | Huilend is het jongetje bij zijn moeder gekomen toen hij uitgescholden werd met het woordje | 22 | Crying has the boy by his mom come when he abused was with the word |
| 130 | Tijdens de viering is er een spetterende lichtshow en vormt het vuurwerk in de lucht het woordje | 22 | During the celebration is there a splattering lightshow and form the fireworks in the air the word |
| 131 | De kleurrijke viooltjes in het bloemenperk voor het drukke winkelcentrum vormen het woordje | 22 | De colorful violets in the flowerbed in front of the crowded shopping center formed the word |
| 132 | Door het storende geluid kon het publiek de tekst niet meer goed horen die volgde op het woordje | 22 | Because of the interfering sound could the audience the text not anymore well hear that followed (on) the word |
| 133 | Uit verdriet om zijn verloren knuffel, zei het kleine jongetje heel lief tegen zijn moeder het woordje | 22 | Out of distress over his lost stuffed animal, said the little boy very sweetly to his mom the word |
| 134 | Het in december gehuwde stel is in juni gescheiden door een ruzie over het woordje | 22 | The in december married couple is in june divorced because of an argument about the word |
| 135 | Bij kinderen opvoeden komt veel geduld kijken, zeker bij het doorzeuren over het woordje | 22 | By children raising comes a lot of patience looking, certainly with the harping on about the word |
| 136 | Hoewel ik tijdens de toets de goede term niet wist, heb ik toch een cirkel gezet om het woordje | 22 | Although I during the test the right term not knew, have I still a circle put around the word |
| 137 | Er liepen mensen weg toen de toneelvoorstelling begon met een korte sketch over het woordje | 22 | There were walking people away when the theatrical performance started with a short sketch about the word |
| 138 | Bij binnenkomst in het mooie centrum zien de toeristen eerst een groot monument voor het woordje | 22 | By entering in the beautiful (city) center see the tourists first a big memorial for the word |
| 139 | De kleine duizendpoot poetst geconcentreerd zijn vele schoenen terwijl hij droomt over het woordje | 22 | The small centipede cleans intently his many shoes while he dreams about the word |
| 140 | De vluchteling vindt het vreemde schrift erg moeilijk en worstelt nu in het bijzonder met het woordje | 22 | The refugee finds the foreign writing very difficult and struggles now especially with the word |
| 141 | De nieuwsbrief die zij 's ochtends ontvingen in het buurthuis stond vol met foto's en tevens het woordje | 22 | The newsletter that they in the morning received in the community center was full of photos as well as the word |
| 142 | In de vernieuwde winkel hingen de ingehuurde medewerkers veel grote posters op met het woordje | 24 | In the renewed store hung the hired employees many big posters up with the word |
| 143 | Het computersysteem werkte niet meer toen er te veel zinnen op het schermpje verschenen met het woordje | 24 | The computer system worked not anymore when there were too many sentences on the screen appeared with the word |
| 144 | Er vloog een bijzonder vliegtuigje over de uitgestrekte weide en die schreef in de lucht het woordje | 24 | There flew a special airplane over the vast meadow and it wrote in the air the word |
| 145 | De onzekere jongen stottert niet veel meer, echter hij heeft enkel nog een klein beetje moeite met het woordje | 24 | The insecure boy stutters not a lot anymore, however he has only a little (bit) difficulty with the word |
| 146 | Er werd een reeks woorden gepresenteerd en de deelnemers moesten toen op een knop drukken bij het woordje | 24 | There was a series (of) words presented and the participants had to push (on) a button at the word |
| 147 | Hij opende de grote dozen die zijn broer die ochtend moest inleveren en riep telkens het woordje | 24 | He opened the big boxes that his brother that morning had to hand in and yelled repeatedly the word |
| 148 | Tijdens het eten spreekt de schoonzus veel over het verleden en vermijdt zo veel mogelijk het woordje | 24 | During (the) dinner speaks the sister-in-law a lot about the past and avoids as much as possible the word |
| 149 | Toen de juf de eerste spellingstoetsen ging corrigeren, zette ze een grote streep onder het woordje | 24 | When the teacher the first spelling tests started correcting, put she a big stripe under the word |
| 150 | De gedurfde tiener won de beruchte wedstrijd met een zeer donkere foto met erop het woordje | 24 | The daring teenager won the notorious competition with a very dark photo with on it the word |
| 151 | Tijdens een Belgische televisiequiz kon de nerveuze deelnemer steeds niet komen op het woordje | 24 | During a Belgian television quiz could the nervous participant constantly not find the word |
| 152 | De boerin schrok zich een hoedje toen ze in de grote winkel keek op het etiketje met het woordje | 24 | The farmer scared herself a hat / was shocked when when she in the big store looked at the label with the word |
| 153 | Zijn vervelende zusjes zingen sinds deze ochtend continu mee met een liedje over het woordje | 24 | His annoying sisters sing since this morning continuously along with the song about the word |
| 154 | Tijdens de heftige discussie over de teksten in de brief hield ze voet bij stuk over het woordje | 24 | During the fierce discussion about the texts in the letter held she stood firm about the word |
| 155 | Toen de bestuurder tegen de overstekende scooter botste schreeuwde hij beledigend het woordje | 24 | When the driver with the crossing scooter collided yelled he offensively the word |
| 156 | Door de snel opkomende duisternis is het voor de mensen niet te doen om uit te kijken voor het woordje | 24 | Because of the quickly coming darkness is it for the people impossible to watch out for the word |
| 157 | Hij probeerde net een kruiswoordpuzzel op te lossen toen zijn moeder hem vertelde over het woordje | 24 | He tried just a crossword puzzle to solve when his mother him told about the word |
| 158 | De politicus bleef onrustig woelen in zijn bed toen hij vorige week hoorde over het woordje | 24 | The politician kept restlessly tossing about in his bed when he last week heard about the word |
| 159 | De dyslectische jongen heeft geen moeite met spelling; echter, hij heeft wel veel problemen met het woordje | 24 | The dyslexic boy has no trouble with spelling; however, he does have a lot of problems with the word |
| 160 | Toen de telefoon over ging, hoorde ze eerst geen geluid uit de hoorn komen en toen enkel het woordje | 24 | When the telephone ringed, heard she first no sound out of the receiver coming and then only the word |
| 161 | Ze speelden een spel met woorden die uitgebeeld moesten worden en bij zijn beurt stond op het scherm het woordje | 24 | They played a game with words that portrayed had to be and at his turn stood on the screen the word |
| 162 | Er zijn niet veel woorden, sommige uitzonderingen niet meegerekend, die klinken gelijk het woordje | 24 | There are not many words, some exceptions not taken into account, that sound like the word |
| 163 | Op het bureau van de dikke directeur stonden kleine beeldjes en grote trofeeën met het woordje | 24 | On the desk of the fat manager stood small statues and big trophies with the word |
| 164 | De slimme professor bedenkt een volledig nieuw experiment om mensen te testen op het woordje | 24 | The clever professor thinks of a completely new experiment to people test on the word |
| 165 | Het Nijmeegse hockeyteam liet hun rood gestreepte wedstrijdshirtjes telkens weer bedrukken met het woordje | 24 | The Nijmegian hockey team let/had their red striped match shirts each time printed with the word |
| 166 | Uiteindelijk werd het kleine jongetje gevonden bij de bekende winkel met op de deur het woordje | 24 | Eventually was the little boy found at the well-known store with on the door the word |
| 167 | Terwijl hij met zijn vriendin koffie dronk, vertelde hij uitgebreid over zijn boek dat ging over het woordje | 24 | While he was with his girlfriend coffee drinking, told he extensively about his book that was about the word |
| 168 | Toen de scholier zijn toets inleverde bij de juf, kon hij niet stoppen met piekeren over het woordje | 24 | When the pupil his test handed in to the teacher, could he not stop with worrying about the word |
| 169 | Het kostte de promovendus heel veel moeite om een uitstekend essay te schrijven over het woordje | 24 | It cost the doctoral student a lot of effort to an excellent review write about the word |
| 170 | Op de snelweg is er deze morgen een ernstig ongeluk gebeurd bij het verkeersbord met het woordje | 24 | On the highway has this morning a severe accident happened at the traffic sign with the word |
| 171 | Sommige mensen zijn weggevoerd wegens extreem geweldgebruik bij de protesten tegen het woordje | 24 | Some people are carried away because of extreme violence at the protests against the word |
| 172 | Om de jongen een beetje te troosten schrijft de vriendelijke dokter op het gips om zijn been het woordje | 24 | To the boy a bit comfort writes the friendly doctor on the cast around his leg the word |
| 173 | De grijze dolfijn springt sierlijk door een hoepel terug in de zee bij het korte bevel beginnend met het woordje | 24 | The grey dolphin leaps gracefully through a ring back in the sea at the short command starting with the word |
| 174 | Het is niet moeilijk om veel leuke en geschikte dichtregels te bedenken die rijmen op het woordje | 24 | It is not difficult to a lot fun and suitable verses think of that rhyme with the word |
| 175 | In het onbewoonde gebied stopte de schipbreukeling een briefje in een fles met erop het woordje | 24 | In the uninhabited area put the shipwrecked person a note in the bottle with on it the word |
| 176 | De politicus die de gemeenteverkiezingen wil winnen weigert te spreken over het woordje | 24 | The politician that the town elections wants to win refuses to speak about the word |
| 177 | In hersenspinsels verzonken over verre oorden en nieuwe culturen peins ik over het woordje | 24 | In fantasies lost about distant places and new cultures ponder I over the word |
| 178 | In het bos bewogen de bomen door de wind en leek het of er een kind foeterde over het woordje | 24 | In the forest moved the trees because of the wind and seemed it like there was a child raging about the word |
| 179 | Het is vervelend voor mensen die niet tegen kletsen kunnen om telkens te horen over het woordje | 24 | It is annoying for people who not can stand chatting to continuously hear about the word |
| 180 | Bij sommige chique gelegenheden is het niet netjes om steeds te beginnen over het woordje | 24 | At some chic occasions it is not decent to keep bringing up the word |
| 181 | Er is tussen docenten op scholen weinig tot geen discussie over spelling met betrekking tot het woordje | 26 | There is between teachers at schools little to no discussion about spelling with regard to the word |
| 182 | Eergisteren vond de populaire kledingontwerper in zijn post een roze folder met erop het woordje | 26 | The day before yesterday found the popular (clothes) designer in his mail a pink leaflet with on in the word |
| 183 | De president uit zijn verontruste gevoelens omtrent de recente ontwikkelingen rondom het woordje | 26 | The president expresses his concerned feelings about the recent developments around the word |
| 184 | Toen de politieknecht de vluchtende crimineel in zijn been schoot, riep hij vloekend het woordje | 26 | When the police servant shot the escaping criminal in his leg, yelled he cursingly the word |
| 185 | Toen de dochter eindelijk nieuwe schoenen kocht, zuchtte de moeder opgelucht en zette een kruis door het woordje | 26 | When the daughter finally new shoes bought, sighed the mother of relief and put a cross through the word |
| 186 | Toen de gemene heks het onschuldige meisje wilde betoveren schreeuwde zij luid een spreuk met het woordje | 26 | When the evil witch the innocent girl wanted to cast a spell on shouted she loudly a spell with the word |
| 187 | Toen ze het mooie gedicht voordroeg vertoonde ze weinig emotie, zelfs niet toen het eindigde met het woordje | 26 | When she the beautiful poem recited showed she little emotion, even not when it ended with the word |
| 188 | Voor het huiswerk voor Engels moesten de leerlingen deze week een zeer kort opstel schrijven over het woordje | 26 | For the homework for English (class) had the children this week a very short essay to write about the word |
| 189 | Voor de sporter begon met de wedstrijd, rende hij richting de tribune en riep toen met luide stem het woordje | 26 | Before the sportsman started with the game, ran he in the direction of the crowd and yelled then with a loud voice the word |
| 190 | De kunstenaars leken tijdens de tentoonstelling tevreden te zijn over de kunstwerken over het woordje | 26 | The artists seemed during the exhibition satisfied to be with the works of art about the word |
| 191 | In het kleine dorpje ontstond er erg veel ophef over de op de muur gespoten tekst met erin het woordje | 26 | In the small village started there a lot of fuss about the on the wall spray-painted text with in it the word |
| 192 | Tijdens het geïmproviseerde bedrijfsuitje, bleven de mensen continu roddelen over het woordje | 26 | During the improvised company outing, kept the people continuously gossiping about the word |
| 193 | Bij de logopediste moest het onzekere meisje drie keer in de week oefeningen doen met het woordje | 26 | At the speech therapist had the insecure girl three times a week exercises to do with the word |
| 194 | Om de geheime poort te openen strijk je met je vinger over het steentje heen en weer en fluister je het woordje | 26 | To the secret gate open stroke you with your finger over the stone back and forth and whisper you the word |
| 195 | Mijn beste vriendin ontving vorige herfst meerdere keren een brief met erop een tekening en het woordje | 26 | My best friend received last fall multiple times a letter with on it a drawing and the word |
| 196 | Tijdens de voorstelling met de leeuwen en tijgers, roept de verzorger in de dierentuin geregeld het woordje | 26 | During the show with the lions and the tigers, shouts the attendant in the zoo regularly the word |
| 197 | Met het oog op de veiligheid wordt er bij een bezoeker onderzocht of hij iets gezegd heeft over het woordje | 26 | Considering the safety is it for every visitor investigated whether he something said about the word |
| 198 | Tijdens de bijeenkomst op het werk hebben de medewerkers de neiging te discussiëren over het woordje | 26 | During the meeting at work have the employees the tendency to argue about the word |
| 199 | In het voorwoord in de bestseller vertelde de succesvolle schrijver geïnspireerd te zijn door het woordje | 26 | In the preface in the best seller said the successful writer inspired to be by the word |
| 200 | Tijdens het wekelijkse uitje vertelde hij voor de zoveelste keer een gedurfde mop over het woordje | 26 | During the weekly outing told he for the umpteenth time a daring joke about the word |

**References**

1. Rouder, J. N., Speckman, P. L., Sun, D., Morey, R. D. & Iverson, G. Bayesian t tests for accepting and rejecting the null hypothesis. *Psychon. Bull. Rev.* **16**, 225–237 (2009).

2. Dienes, Z. Using Bayes to get the most out of non-significant results. *Front. Psychol.* **5**, 1–17 (2014).
